# CDH4 suppresses the progression of salivary adenoid cystic carcinoma via E-cadherin co-expression

**DOI:** 10.18632/oncotarget.12821

**Published:** 2016-10-22

**Authors:** Jian Xie, Yan Feng, Ting Lin, Xiao-Yu Huang, Rui-Huan Gan, Yong Zhao, Bo-Hua Su, Lin-Can Ding, Lin She, Jiang Chen, Li-Song Lin, Xu Lin, Da-Li Zheng, You-Guang Lu

**Affiliations:** ^1^ Department of Preventive Dentistry, Affiliated Stomatological Hospital, Fujian Medical University, Fuzhou, China; ^2^ Department of Pathology, Affiliated Stomatological Hospital, Fujian Medical University, Fuzhou, China; ^3^ Center of Dental Implant, Affiliated Stomatological Hospital, Fujian Medical University, Fuzhou, China; ^4^ Department of Oral and Maxillofacial Surgery, Affiliated First Hospital of Fujian Medical University, Fuzhou, China; ^5^ Key Laboratory of Ministry of Education for Gastrointestinal Cancer, School of Basic Medical Sciences, Fujian Medical University, Fuzhou, China

**Keywords:** CDH4, salivary adenoid cystic carcinoma, CDH1, proliferation, invasion

## Abstract

The cadherin-4 gene (CDH4) of the cadherin family encodes non-epithelial R-cadherin (R-cad); however, the function of this gene in different types of cancer remains controversial. In this study, we found higher expression of CDH4 mRNA in a salivary adenoid cystic carcinoma (SACC) cell line with low metastatic potential (SACC-83) than in a cell line with high metastatic potential (SACC-LM). By analyzing 67 samples of SACC tissues and 40 samples of paraneoplastic normal tissues, we found R-cad highly expressed in 100% of normal paraneoplastic tissue but only expressed in 64% of SACC tumor tissues (P<0.001). Knockdown of CDH4 expression in vitro promoted the growth, mobility and invasion of SACC cells, and in vivo experiments showed that decreased CDH4 expression enhanced SACC tumorigenicity. Furthermore, CDH4 suppression resulted in down-regulation of E-cadherin (E-cad), which is encoded by CDH1 gene and is a well-known tumor suppressor gene by inhibition of cell proliferation and migration. These results indicate that CDH4 may play a negative role in the growth and metastasis of SACC via co-expression with E-cadherin.

## INTRODUCTION

Salivary adenoid cystic carcinoma (SACC) is a common malignant salivary gland tumor that is strongly invasive and has high rates of relapse, metastasis and mortality. As the 10-year survival rate for patients with SACC is only 29%-40% following surgery and postoperative radiotherapy [1], it is necessary to identify genes associated with SACC invasion and metastasis and to clarify their functions. Such efforts may reveal target genes for the prevention and treatment of SACC and for improving the long-term survival and quality of life of patients.

Cadherins, which have been detected in more than thirty species, are calcium-dependent proteins present in various parts of the body that mediate cell-cell adhesion via homo- or heterotypic interactions. In addition to cell-cell adhesion, the cadherin structure suggests that these proteins play a key role in building higher organizational structure [2–4]. Cadherins have also been linked to intracellular signaling, such as the WNT, EMT and FGF pathways [5–7]. Moreover, mounting evidence suggests that the cadherin family plays important roles in tumorigenesis, invasion, and metastasis [8–10].

Research into the relationship between cadherin and adenoid cystic carcinoma is ongoing. Some studies have found that E-cadherin is down-regulated in SACC compared to normal and adenoid tissues and that E-cadherin down-regulation may promote nerve invasion, lymphatic and regional recurrence and distant metastasis [11, 12]. Zhang et al. reported that expression levels of E-cadherin-catenin are positively correlated with the degree of SACC cell differentiation [13]. Wang JF et al. found that N-cadherin was abnormally expressed in highly metastatic SACC tissue, promoting invasion and migration in SACC cells [14]. Although evidence on the relationship between cadherin family genes and SACC is increasing, the role of the cadherin-4 gene (CDH4) in SACC remains unknown.

In this study, we investigated the role of CDH4 in SACC and found that this gene inhibited the proliferation, invasion and migration of SACC in vitro and suppressed tumorigenicity in vivo. Moreover, we found that CDH4 impeded the progression of SACC, as its expression was positively correlated with CDH1. Our results suggest that CDH4 might function as a tumor suppressor gene.

## RESULTS

### CDH4 expression is reduced in clinical SACC samples

**Figure 1:**
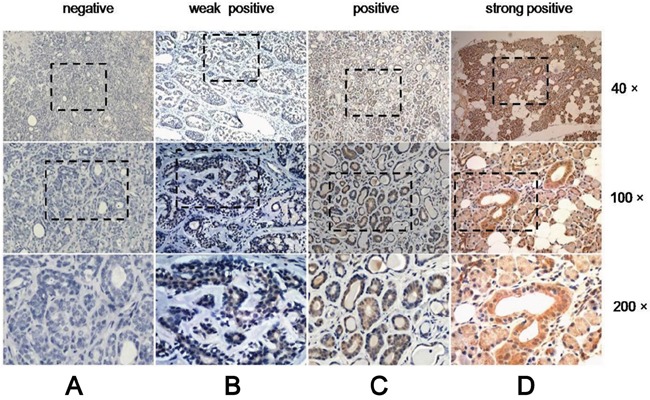
Expression of CDH4 in SACC is lower than in normal tissue Representative images for negative, weakly positive and positive expression of CDH4 in SACC tissues **(A-C)** and strongly positive expression in normal tissue **(D).**

To elucidate the role of CDH4 in SACC, we examined its expression by immunohistochemistry in 67 samples of SACC and 40 samples of paraneoplastic normal tissues, which served as the control group. Of the 67 samples of SACC tissues, R-cad was only expressed in 40 samples, whereas all 40 samples in the control group expressed R-cad. As shown in Figure 1, expression of CDH4 was significantly higher in paraneoplastic normal tissues than in SACC tissues (P<0.001, Table 1). Furthermore, we examined whether CDH4 levels are related to clinical feature of SACC. As shown in Table 2, the expression of CDH4 was lower in the tumors with late stage (stage III/IV) than that with early stage (stage I/II, P=0.01). These results indicated that CDH4 may play a suppressive role in SACC.

**Table 1:** The expression of CDH4 in tissues of normal salivary and SACC cases

Samples	Cases	Negative	Weakly positive	Positive	Strongly positive	P-value
Normal Salivary	40	0	0	13	27	<0.001
SACC	67	24	29	14	0	

Rank-sum test Z=-8.309.

**Table 2:** The expression of CDH4 and CDH1 in clinical and pathological characteristics of SACC

Characteristics	For CDH4					For CDH1			
		Total	Low CDH4*	High CDH4*	P value	Total	Low CDH1	High CDH1	P value
Gender									
	Female	39	31	8	1.00	16	13	3	0.23
	Male	28	22	6		14	8	6	
Age									
	≤55	38	27	11	0.07	16	12	4	0.69
	>55	29	26	3		14	9	5	
Stage									
	Early	31	20	11	0.01**	12	7	5	0.41
	Late	36	33	3		18	14	4	
Invasion									
	No	28	21	7	0.55	11	7	4	0.69
	Yes	39	32	7		19	14	5	
Metastasis									
(Lymph node and distant)	No	51	38	13	0.16	25	17	8	1.00
	Yes	16	15	1		5	4	1	

* Because of limited samples number, the expression of CDH4 and CDH1 was divided into two levels, in which low expression included the Negative and weakly positive as shown in Table 1 and 3, and high expression included positive and strongly positive.

** P<0.05.

### Knockdown of CDH4 promotes SACC cell proliferation in vitro

**Figure 2:**
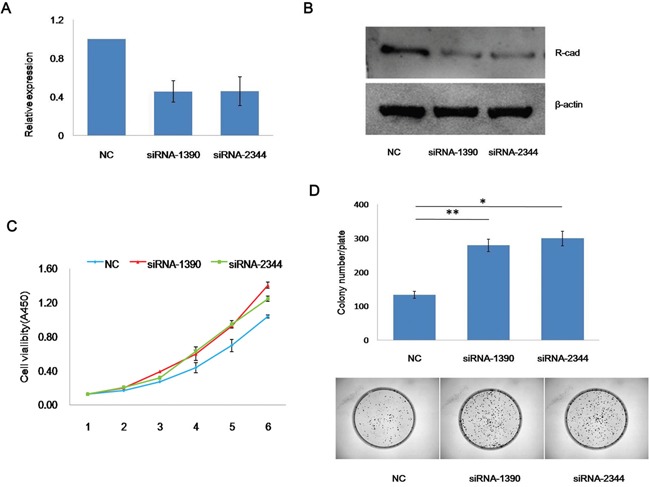
Knockdown of CDH4 promotes the proliferation of SACC-83 cells **(A-B)** CDH4 targeting siRNAs effectively reduced CDH4 expression as measured by real-time PCR (A) and western blot (B). **(C)** After transfected with CDH4 siRNAs and NC, the growth curves of SACC-83 cells were measured by CCK-8 reagents (P<0.01 at days 3, 4 and 5). **(D)** The proliferation of SACC-83 cells after CDH4 knockdown was detected by colony formation. The number of colonies was counted (P<0.05). The experiment was repeated three times.

To investigate the function of CDH4 in cancer cell proliferation, siRNAs targeting CDH4 (siRNA-1390, siRNA-2344) were transfected into SACC-83 cells to knockdown CDH4 expression. Compared with the negative control group (NC), the expression of CDH4 was significantly reduced, as shown by real-time PCR (Figure 2A) and western blotting (Figure 2B). According to results of the CCK-8 assay (Figure 2C, P<0.01 at days 3, 4 and 5) and a colony formation assay (Figure 2D, P<0.05, n=3), knockdown of CDH4 gene expression promotes SACC-83 cell proliferation.

### CDH4 suppresses SACC migration and invasion in vitro

**Figure 3:**
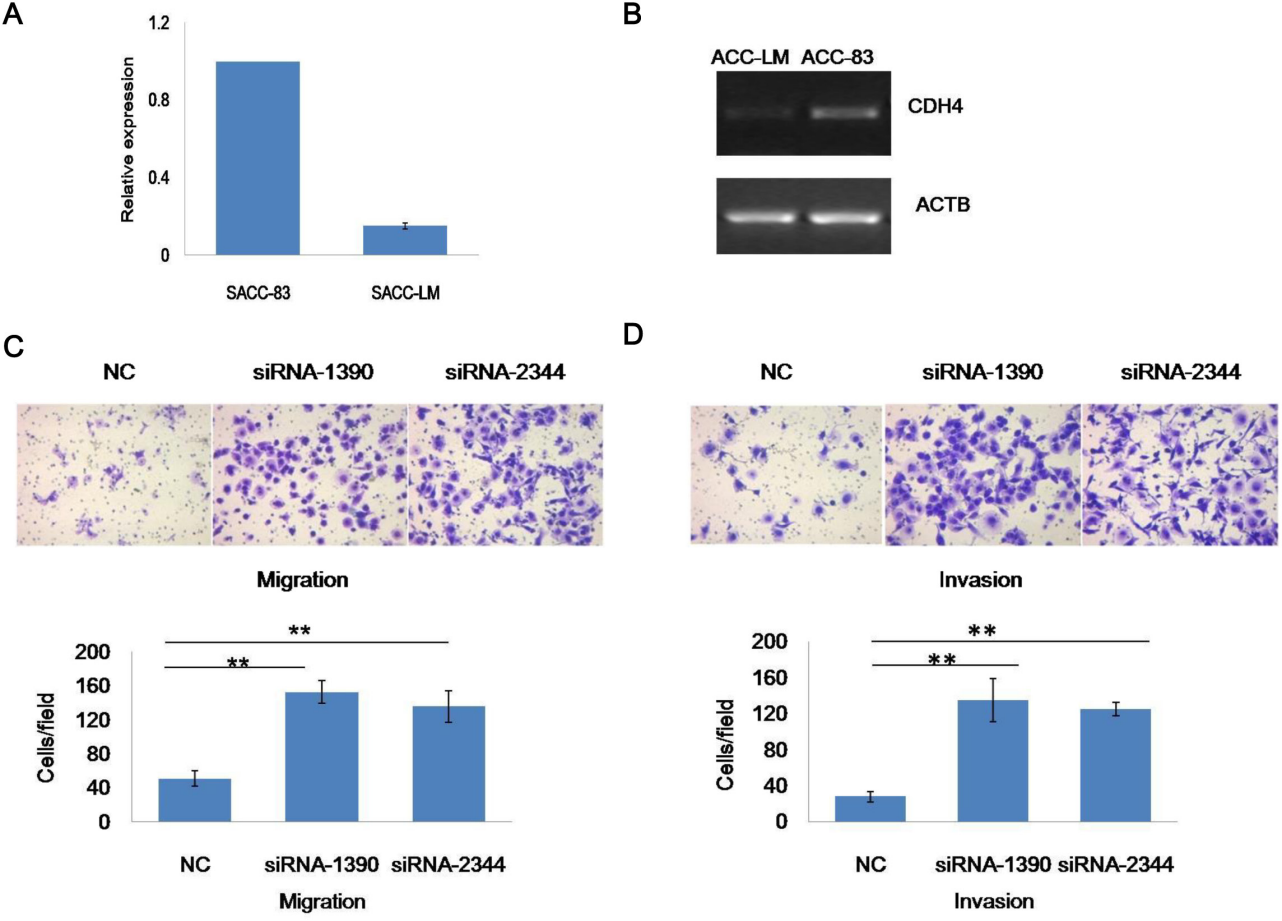
CDH4 is associated with cell mobility of SACC **(A-B)** Expression of CDH4 in SACC-83 cells was higher than in SACC-LM cells according to real-time RT-PCR (A) and Sq-RT-PCR (B). **(C)** Representative images of cells transfected with CDH4 siRNAs and the NC group subjected to a Transwell assay without (upper panel) Matrigel coating. The number of cells migrating through the filters was counted. **(D)** Representative images of cells transfected with CDH4 siRNAs and the NC group subjected to a Transwell assay with (upper panel) Matrigel coating. The number of cells invading the filters was counted. The numbers of migrating and invading cells are presented as mean values from at least five randomly selected low-power fields (100×) from three independent experiments. P<0.01 when compared with the control (NC).

To explore the roles of CDH4 in metastasis of SACC, quantitative reverse transcription polymerase chain reaction (qRT-PCR) and semi-quantitative RT-PCR were used to assess expression in SACC cell lines SACC-83 and SACC-LM. The results indicated up-regulation of CDH4 in the low-metastatic cell line SACC-83 compared with the high-metastatic cell line SACC-LM (Figure 3A and 3B, P<0.05, n=3), which indicated that CDH4 may associate with SACC metastasis.

Next cell invasion and cell migration assays were performed to further determine the roles of CDH4 in the control of cell mobility. In the cell migration assay, the number of cells passing through monolayers in the siRNA-1390 (152.78±13.60) and siRNA-2344 (135.78±18.50) groups was higher than in the NC group (51.22±9.00; Figure 3C; P<0.01, n=3). In addition, the number of cells able to migrate through Matrigel was higher in the siRNA-1390 (134.89±24.10) and siRNA-2344 (125.00±7.40) groups than in the NC group (27.78±5.90; Figure 3D; P<0.01, n=3). Overall, down-regulation of CDH4 expression significantly improved the migration and invasion of SACC-83 cells, confirming that CDH4 may contribute to SACC cell migration and invasion.

### Knockdown of CDH4 promotes tumorigenicity of SACC cells in vivo

**Figure 4:**
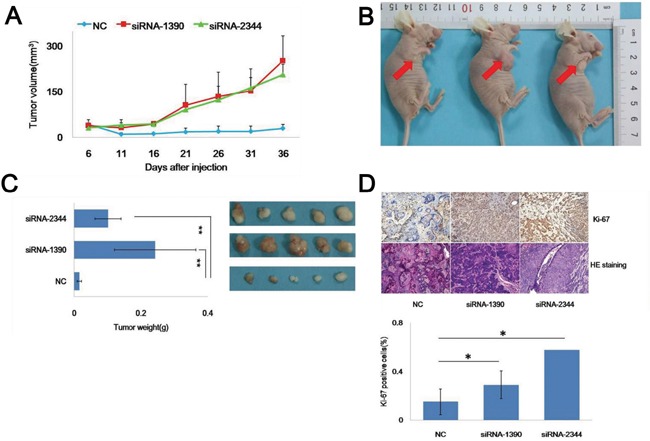
Knockdown of CDH4 promotes SACC-83 cell tumorigenicity in vivo **(A)** Growth curves of tumors formed by cells of the CDH4-knockdown groups and the NC group. Tumors in the flank of nude mice were measured with a digital caliper every 5 days for 36 days. **(B)** Representative images of tumors on the flanks of nude mice are shown. **(C)** After 36 days, the tumors were excised, photographed and weighed. **(D)** Expression of Ki-67 (upper part) and cell differentiation (lower part) were detected in the xenograft tumors using immunohistochemistry (DAB, 40×) and HE staining (40×). The chart shows Ki-67 positivity.

We then constructed a xenograft model in vivo to address the oncogenic effect of CDH4 in tumorigenicity. The growth curves generated showed that tumors formed in the siRNA-1390 and siRNA-2344 groups on day 6 after inoculation and that the velocity of tumor growth at every observation point in the knockdown groups was higher than in the NC group (Figure 4A). Tumor formation and changes in nodule volume in the three groups are presented in Figure 4B. After 38 days, the volume and wet weight of the tumors in the siRNA-1390 (253.12±82.01) and siRNA-2344 (206.84±33.94) groups were greater than in the NC group (25±3.77; Figure 4C; P<0.05, n=5). We next assessed expression of Ki-67 in xenograft tumor tissue using immunohistochemistry and observed tissue differentiation with HE staining (Figure 4D). After CDH4 knockdown in SACC-83 cells, the percentage of proliferating cells increased, and the degree of differentiation of carcinoma decreased. The results suggest that the decline of CDH4 in SACC-83 cells may have promoted cell growth by reducing differentiation.

### Expression levels of CDH4 and CDH1 are positively correlated in both cells and clinical salivary adenoid cystic carcinoma samples

**Figure 5:**
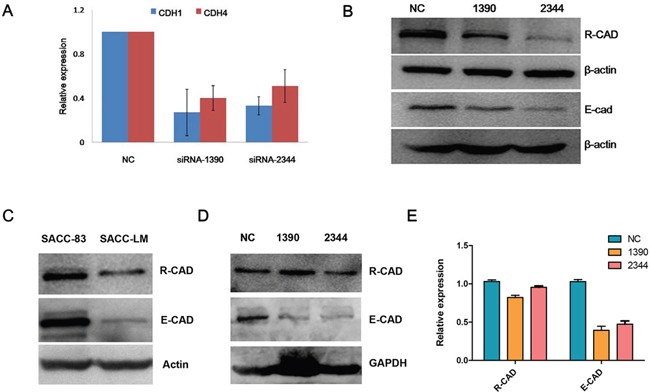
Expression levels of CDH4 and CDH1 are positively correlated in cells **(A-B)** After knockdown of CDH4, the expression of CDH1 was detected by real-time PCR (A) and Western blot (B). **(C)** The expression levels of CDH1 and CDH4 in SACC-83 and SACC-LM were detected by Western blot. **(D-E)** The expression of CDH4 and CDH1 in xenograft tumors was detected by Western blot (D) and the relative expression was quantified and normalized to GAPDH. (P>0.05 for CDH4 and P<0.05 for CDH1).

To determine the mechanism by which CDH4 suppresses the progression of SACC, E-cad transcription and translation was evaluated when expression of CDH4 was reduced. Real-time PCR and western blotting revealed that E-cad mRNA (Figure 5A) and protein (Figure 5B) expression was down-regulated when CDH4 expression was down-regulated in SACC-83 cells. To exclude the possible off-target effect of siRNAs, we checked the specificity of these two siRNAs by BLAST and alignment using DNAMAN. The max complementarity of these 2 siRNAs with CDH1 mRNAs in discontinuous is only 12 bp and the max complementarity in continuous is only 7 bp. Also, the expression pattern of CDH4 and CDH1 in SACC-83 and SACC-LM cells is similar (Figure 5C). In the xenograft tumors, the expression of CDH4 in siRNA-1390 and siRNA-2344 groups was similar to NC, but the expression of CDH1 was still lower than NC groups in the time point of 38 days after siRNAs transfection (Figure 5D and 5E, P>0.05 for CDH4 and P<0.01 for CDH1).

**Figure 6:**
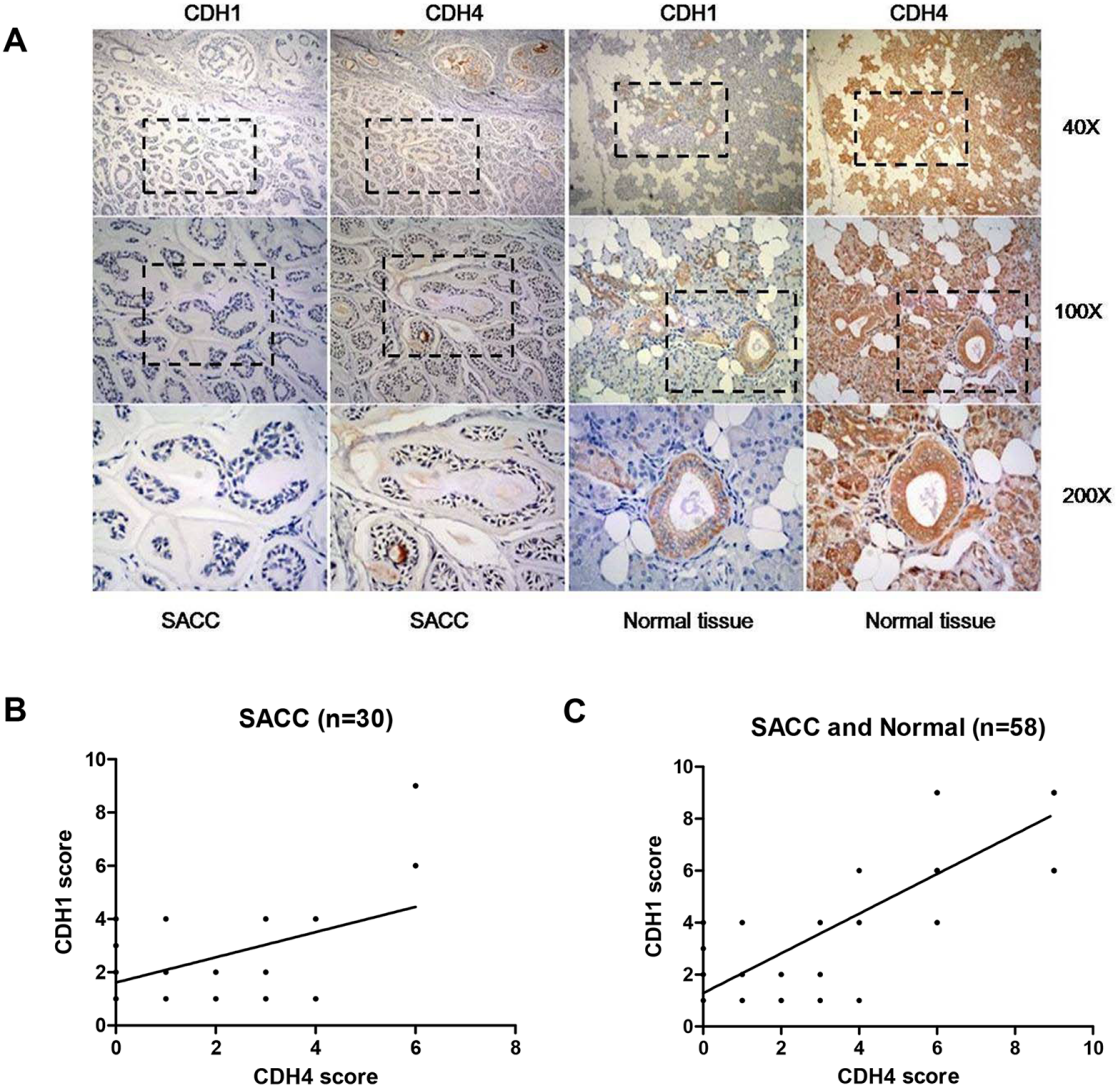
Expression levels of CDH4 and CDH1 are positively correlated in clinical SACC samples **(A)** Representative images for CDH1 and CDH4 in the same tissues. **(B-C)** The correlation of the expression of CDH1 and CDH4 when only SACC tissues were included (B, n=30, R^2^=0.22, P<0.01) or when both SACC and normal tissues were included (C, n=58, R^2^=0.76, P<0.0001).

Furthermore, we assessed expression of CDH1 and CDH4 in the same SACC and paraneoplastic normal salivary tissues (Table 2) using immunohistochemistry. As shown in Table 3 and Figure 6A, expression of E-cad in paraneoplastic normal tissues was significantly higher than that in SACC tissues (P<0.01). Furthermore, the expression score of CDH4 and CDH1 in the same tissues were positively correlated when only the 30 SACC tissues were included (Figure 6B, n=30, P<0.01) or when both SACC tissues and normal salivary were considered (Figure 6C, n=58, P<0.0001).

**Table 3:** The expression of CDH1 in tissues of normal salivary and SACC cases

Samples	Cases	Negative	Weakly positive	Positive	Strongly positive	P-value
Normal Salivary	28	0	0	11	17	<0.01
SACC	30	0	21	8	1	

Rank-sum test Z=-5.888.

## DISCUSSION

Retinal cadherin (R-cad), which is encoded by the CDH4 gene, is a member of the cadherin family. Previous studies found that R-cad functions in the development of normal retinal, brain, muscle, gastrointestinal, pancreas and kidney tissues [15–17]. However, the function of R-cad in cancer is controversial because its expression was found to be both up-regulated and down-regulated in some cancers and differential expression of R-cad has been detected in some cancer cell lines [18–21]. In addition, R-ad has been reported to either promote or suppress tumor genesis and metastasis, with some studies showing that R-cad plays a positive role in cancer progression [22–24]. These reports suggest that R-cad promotes the migration of cells via Rho GTPase activation [22]. R-cad has also been shown to compete with E-cad for P120 and to promote the migration of A431 cells [23]. Although R-cad expression has been found in rhabdomyosarcomas, it was absent in normal myoblasts [24]. Conversely, R-cad acts as a suppression factor in other tumor types. Previous studies indicate that R-cad expression usually diminishes with cancer progression in tumorigenic cell lines via methylation or down-regulation; such changes have been detected in mammary tumors [20], gastric carcinomas [21], nasopharyngeal carcinoma [25] and colorectal cancer [26]. Moreover, R-cad was found to inhibit expression of MMP-1, MMP-2, and Cox-2 in mammary tumors [20].

Our data suggest that R-cad is down-regulated in SACC tissues compared with paraneoplastic normal tissues and that R-cad mRNA is more highly expressed in SACC cell lines with low metastatic potential (SACC-83) compared with highly metastatic cell lines (SACC-LM). We found that knockdown of CDH4 not only improved the mobility and invasion of SACC-LM cells in vitro but also promoted the growth of SACC-LM cells in vitro and tumorigenicity in vivo. These results suggest that CDH4 might play a negative role in SACC.

Encoded by the CDH1 gene, E-cad is an important cell adhesion molecule that mediates tight binding to epithelial cells [27]. In recent years, many studies have shown that E-cad inhibits tumor invasion and metastasis [28–32]. Increasing evidence has proven that E-cad not only suppresses the invasion and metastasis of cancer cell lines but also suppresses their growth [33]. E-cad has also been considered to function as a tumor suppressor in SACC. As E-cad and R-cad are members of the same family, they share a common structure and are functionally interrelated [34]. E-cad [35, 36] and R-cad [20, 21] also function in epithelial maintenance and are readily methylated in cancer tissues, and research has shown that the expression levels of E-cadherin-catenin are positively correlated with the degree of SACC cell differentiation. Our data revealed that R-cad is positively correlated with the degree of differentiation of SACC. There may be an association between E-cad and R-cad; moreover, a low level of CDH4 expression led to the down-regulation of E-cad.

To validate our hypothesis, we used real-time PCR, immunohistochemistry and immunoblotting in SACC and tongue cancer cell lines. The results of these experiments confirm that knockdown of CDH4 expression results in down-regulation of both E-cad mRNA and protein, and our immunohistochemical analysis showed R-cad expression to be positively correlated with E-cad expression in SACC tissues. We suggest that R-cad suppresses the growth and metastasis of SACC due to changes in the expression of E-cad.

The relationship between R-cad and E-cad in A431 cells is different from that in SACC [37]. Recent studies have proven that the adhesion activity of cadherins is controlled in a cell context-dependent manner [38], promoting cell mobility in some cell lines while suppressing cell mobility and proliferation in others. The basis for this mechanism of R-cad in SACC and whether it is due to the cellular context of SACC itself remain to be determined. Similarly, why the decreased expression of R-cad induces E-cad down-regulation remains unknown, as do the mechanisms of interaction between CDH1 and CDH4. Further studies will be necessary to clarify these issues.

## MATERIALS AND METHODS

### Tissue specimens

Sixty-seven cases of clinical specimens of SACC were collected between 2004 and 2014 by the Affiliated Union Hospital of Fujian Medical University and Fuzhou General Hospital of Nanjing Military Command. All of these recruited patients underwent radical surgery without preoperative chemotherapy or radiotherapy, and the collected samples were reconfirmed by two pathologists. The project and protocols for the investigation involving human and animal tissues were approved by the ethics committee of Fujian Medical University. Written informed consent was obtained from all patients.

### Cell culture

The high- and low-metastasis SACC cell lines (SACC-LM and SACC-83) were obtained from Peking University Health Science Center. The cells were grown in 1640 culture medium (Gibco BRL, Grand Island, NY) containing 15% fetal bovine serum (Gibco) in a 37°C incubator with humidified air containing 5% CO_2_. Cells in logarithmic growth phase were used for analyses.

### Semi-quantitative reverse transcription PCR (SqRT-PCR) and real-time quantitative RT-PCR (qPCR)

TRIzol Reagent (Invitrogen, USA) was used to extract total RNA from 5×10^5^ cells of the SACC-83 and SACC-LM cell lines growing in six-well plates. The total RNA was reverse transcribed to cDNA using the PrimeScript™ RT reagent kit (Takara, Japan). The 50-μl PCR mixture contained 2 μl cDNA, 10 μl forward primer, 10 μl reverse primer, 4 μl dNTPs, 5 μl 10×PCR buffer, 1 μl Taq polymerase, and 18 μl ddH_2_O. The reaction included denaturation at 98°C for 2 min, followed by 30 cycles of denaturation at 98°C for 30 sec, annealing at 55°C for 30 sec, and extension at 72°C for 3 min. β-Actin (ACTB) was used as an internal control. The reaction products were electrophoresed on a 1% agarose gel and observed under UV light. QPCR was performed using SYBR-Green-PCR Master Mix (Takara, Japan). RNA samples were diluted to the same concentration based on the absorbance at 260 nm for reverse transcription of target cDNA using a PrimeScript™ RT reagent kit (Takara, Japan). ACTB was used as an internal control. The primers used for ACTB, CDH4 and CDH1 are shown in Table 4. The reaction was as follows: denaturation at 95°C for 2 min, followed by 40 cycles at 95°C for 15 sec, 60°C for 30 sec and 95°C for 15 sec. The fluorescent signal was measured at the end of the annealing phase of every cycle.

**Table 4:** The primers for real-time PCR and semi-quantitative RT-PCR in this study

Gene	Accession no.	Forward	Reverse
ACTB	NM 001101	CCTGGCACCCAGCACAAT	GGGCCGGACTCGTCATACT
CDH1	NM 004360.3	GGATGTGCTGGATGTGAATG	CACATCAGACAGGATCAGCAGAA
CDH4	NM001794.2	CGTCCATCATCAAAGTCAAGGT	GGTCGTAGTCCTGGTCCTCCT

### Immunohistochemistry

SACC pathological tissues were stained and analyzed according to an immunohistochemical SP three-step approach. SACC paraneoplastic normal tissue was used as a control group. After deparaffinization and rehydration, antigens on sections were retrieved by boiling in 10 mM sodium citrate buffer (pH 6.0) for 10 min. The sections were incubated in methanol containing 3% H_2_O_2_ for 10 min to restrain endogenous peroxidase activity. After several washes in PBS, the sections were blocked with a universal blocking reagent (Maxin, USA) for 10 min at room temperature. The sections were then incubated with a primary antibody against CDH4 (1:100, Abnova, USA), Ki-67 (1:500, Abcam, UK), or E-cad (1:100, Abcam, USA) for 1 h at room temperature. After several washes in PBS, the sections were incubated with a biotin-conjugated secondary antibody (Maxin) for 10 min at room temperature and then rinsed with PBS. The antibody complexes were visualized by incubation with diaminobenzidine tetrahydrochloride (DAB) chromogen (Maxin). The sections were counterstained with hematoxylin (Dako, Denmark), dehydrated, and examined by light microscopy. All slides were reviewed independently by two pathologists who were blinded to each other's readings. The results of staining were assessed on a four-tier scale: negative, no staining; 1+, weakly positive staining; 2+, positive staining; 3+, strongly positive staining. The percentage of cells stained was divided into the following four levels: no staining, 0; staining area ≤30%, 1; 30% <staining area ≤50%, 2; staining area >50%, 3. The immunohistochemical results were graded according to the product of the above two scores, as follows: 0, negative staining; 0 <score ≤3, weakly positive staining; 3 <score ≤6, positive staining; 6 <score ≤9, strongly positive staining.

### Design and synthesis of corresponding siRNAs

To target different coding regions of CDH4 and CDH1, four corresponding siRNAs were designed and synthesized (Shanghai GenePharma Co, Shanghai, China). The sequence information is presented in Table 5. All siRNAs were separately transfected into SACC-83 cells using Lipofectamine® RNAiMAX Transfection Reagent (Invitrogen, USA) following the manufacturer's instructions.

**Table 5:** The siRNA sequences for CDH4 and negative control

Name	Sequence	
siRNA-1390	5'-CAGUCGACUACGAGCUCAATT-3′	5'-UUGAGCUCGUAGUCGACUGTT-3′
siRNA-2344	5'-GCGACAACAUCCUCAAGUATT-3′	5'-UACUUGAGGAUGUUGUCGCTT-3′
NC	5'-UUCUCCGAACGUGU CACGUTT-3′	5'-ACGUGACACGUUCGGAGAATT-3′

### Western blotting

Total protein (30 μg) was separated by 10% SDS-PAGE and transferred to PVDF membranes (Amersham Biosciences). The membranes were blocked in 1% bovine serum albumin and incubated with primary antibodies against R-cad at a dilution of 1:100 at 4°C for 10 h (Abnova, USA), β-actin at a dilution of 1:1000 at 4°C for 2 h (Santa Cruz, USA), and E-cad at a dilution of 1:1000 at 4°C for 10 h (Abcam, USA). After washing with TBST, alkaline phosphatase-conjugated secondary antibodies were added, and the protein bands were visualized using CDP-Star reagents (Roche, IN, USA).

### In vitro cell proliferation assay

Cell proliferation was evaluated using cell counting and colony formation assays. The cell counting assay included six groups of wells in triplicate and was performed in a 96-well dish. Cells were trypsinized and plated at a rate of 1 × 10^3^ per well. The wells were monitored daily. After the cells adhered, the medium was replaced with 10 μl CCK-8 reagent and 90 μl 1640 without FBS and incubated for 1 h. Absorption was measured at 450 nm. Measurements were taken at the same time of day for the final 5 days, and a graph of cell proliferation was generated. Colony formation was measured in 6-cm plates. Each plate was seeded with 1 × 10^3^ cells, and 8 ml 1640 culture solution containing 15% FBS was added. After 2-3 weeks, the medium was discarded, and the colonies were stained with crystal violet. The dishes were scanned (GE Healthcare, USA), and the number of cells at the bottom of the dishes was counted. The experiments were conducted in triplicate and repeated three times.

### In vitro cell invasion and migration assay

To evaluate variation in SACC-83 cell invasion due to a reduction in CDH4 expression, cell invasion assays were performed using 24-well Transwell chambers coated with Matrigel (8-μm pore size, BD Sciences, USA). The cells were starved in serum-free medium overnight and then collected in 1640 containing 0.1% FBS. A cell suspension of 7 × 10^4^ cells and 500 μl of 1640 containing 0.1% FBS was added to the upper chamber, and 700 μl of 1640 containing 10% FBS was added to the lower chamber. After 48 h, the Matrigel and cells in the upper chamber were removed with cotton swabs. The cells that had migrated through the Matrigel on the lower surface of the membrane were stained with crystal violet. Cells selected randomly from at least five microscopic fields (at 100×) were counted and photographed.

Using a method similar to the invasion chamber and 5×10^4^ cells, a Transwell assay was performed without Matrigel (8-μm pore size, BD Sciences, USA) to examine variation in SACC-83 cell migration. The cells were cultured in 1640 culture medium with 0.1% FBS for 36 h, and we used a method similar to that described above to stain, count and photograph the cells that migrated through the membrane.

### Tumor formation in an animal model

An in vivo cell proliferation assay was applied to assess tumorigenicity in nude mice. Four-week-old BALB/C female nude athymic mice (average weight 20 g) were obtained from the Fujian Medical University animal central laboratory. These mice were fed for 5 days under specific pathogen-free conditions to allow them to adapt to the environment of the central laboratory. SACC-83 (3 × 10^6^) cells in a 0.2 ml cell suspension were injected subcutaneously into the right flank, and the mice were observed carefully every three days. The tumor volume was calculated as V=AB^2^/2, in which A is the maximum diameter and B is the diameter perpendicular to the line of A.

### Statistical analysis

SPSS v22 was used for statistical analyses. Data are expressed as the means ± SD. The rank sum test and the Mann-Whitney U test were applied for statistical analysis of immunohistochemistry data. Differences among groups were compared using one-way analysis of variance (ANOVA). Associations between R-cad and E-cad in samples were analyzed using the Pearson test. P<0.05 was considered statistically significant.
